# Clinical and radiological results of kyphectomy and sliding growing rod surgery technique performed in children with myelomeningocele

**DOI:** 10.1186/s13018-020-02099-2

**Published:** 2020-12-01

**Authors:** Çağrı Özcan, Ömer Polat, İbrahim Alataş, Savaş Çamur, Necdet Sağlam, Bekir Yavuz Uçar

**Affiliations:** 1 University of Health Sciences, Umraniye Education and Research Hospital Department of Orthopaedics and Traumatology, Elmalikent mh34764 Adem yavuz Street No. 1, Umraniye/Istanbul, Turkey; 2 Bilim University Sisli, Florance Nightingale Hospital Department of Neurosurgery, Hüseyin Cahit Yalcin Street no. 1, İstanbul/Besiktas, Turkey

**Keywords:** Myelomeningocele, Kyphectomy, Sliding growing rod, Kyphosis

## Abstract

**Background:**

The aim of this study was to present clinical and radiological results of myelomeningocele (MMC) patients treated with the sliding growing rod (SGR) technique after kyphectomy.

**Methods:**

Between 2016 and 2019, 30 patients (21 males and nine females) who underwent the SGR technique with kyphectomy and posterior instrumentation due to MMC were retrospectively reviewed. Patients’ pre- and postoperative kyphosis, scoliosis, correction rates, bleeding during surgery, blood supply during and after surgery, operation time, instrumentation levels, number of vertebrae removed, MMC onset levels, hospital stay, annual lengthening amounts, and complications were evaluated.

**Results:**

The mean patient age was 6.9 (4–10) years. Mean preoperative kyphosis was 115° (87–166°), mean early postoperative kyphosis was 3.9° (20–10°), and final follow-up postoperative kyphosis was 5.1° (22–8°). In nine patients presenting with scoliosis, scoliosis was evaluated as 60.2° (115–35°) preoperative, as 12.9° (32–0°) early postoperative, and 15.7° (34–0°) in the final measurement. The kyphotic deformity correction rate was 96.5%, and the scoliotic deformity correction rate was 74.9%. A statistically significant difference was seen between pre- and early postoperative values in kyphosis and scoliosis measurements (*p* < 0.05). The annual prolongation of the patients was calculated as averages of 0.72 and 0.77 cm/year between T1–T12 and T1–S1, respectively.

**Conclusion:**

Kyphectomy performed during the early MMC period patients appears to be an excellent method for facilitating rehabilitation and daily care of these patients. It appears that the SGR technique, which provides lung volume protection and lengthening with kyphectomy, is a safe and reliable method in patients.

**Level of evidence:**

Level 4

## Introduction

Spinal deformities are common in patients with myelomeningocele (MMC). Kyphosis occurs in 10 to 20% of MMC patients [1, 2]. Due to kyphosis development, these patients cannot lie on their backs. Balance while sitting is completely disturbed, pelvic obliquity increases over time, and as abdominal pressure increases, these patients may undergo many systematic problems, such as eating imbalances and difficulty in urination [3]. These patients have to sit on their sacrum instead of the ischial tubercle. If this deformity is not corrected, it increases over time. The kyphosis of these patients increases by 4° to 11° yearly [4]. In later periods, severe ulcerations and skin wounds develop in the kyphosis apex. These wounds can progress to osteomyelitis. These patients have to use their hands to maintain truncal balance and increase thoracic volume [3]. If these patients are not treated, they lose free use of their upper extremities. With the increase in kyphosis, the viewing angles increase to the ground rather than across the ground.

The most suitable age and fusion method for kyphectomy in MMS patients is via one of the discussed methods in the literature [5–8]. Surgery in the early period allows these patients to live more comfortably in their older ages. A spine that can be corrected with an easier kyphectomy at an early age may be appealing to the surgeon. However, a fusion to be made during this period may stop the lengthening and produce a small chest cavity [2]. To prevent this process, a short segment posterior instrumentation may be applied to provide fusion after kyphectomy [9–11]. However, a short segment fusion is an important reason for implant failure in these patients. Revision surgery is more difficult due to implant failure. Wound infections and revision surgery can cause major problems [12].

Harrington rods, fusion with plate and screws, Dunn–Mccarthy fixation, and Warner–Fackler techniques are frequently used surgical techniques for kyphectomy in MMC patients [2, 6]. However, lengthening and implant failures have always been the two issues discussed in these patients. In a long segment fusion performed by extending the moment arm, the failure rate decreases; in addition, lengthening and lung volume also decreases [7].

The aim of this study was to examine clinical and radiological results from patients who underwent the sliding growing rod (SGR) technique after kyphectomy in MMC patients and to evaluate the resulting complications.

### Surgical technique pearls

**Fig. 1:**
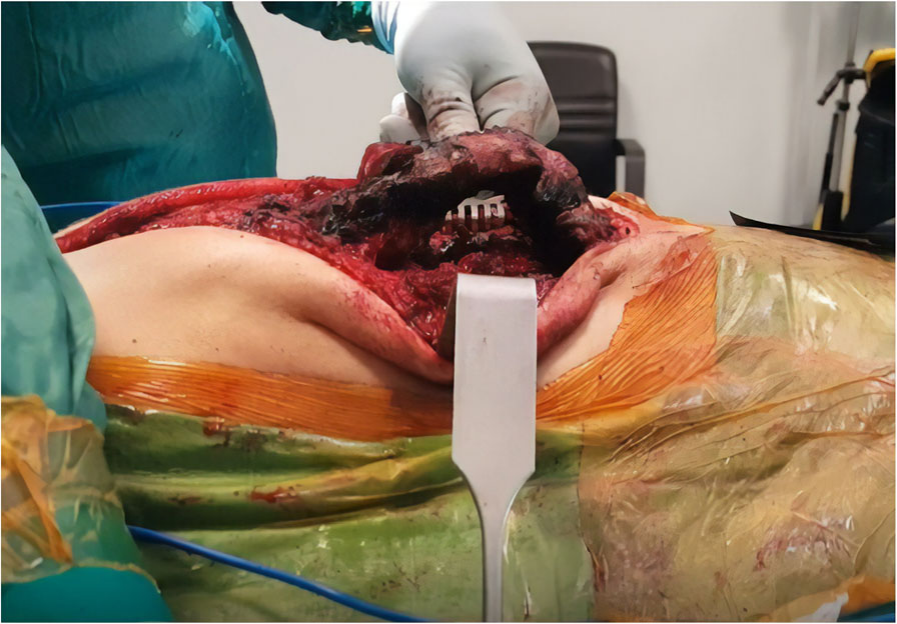
Preparation of the lumbar kyphosis region for kyphectomy

**Fig. 2:**
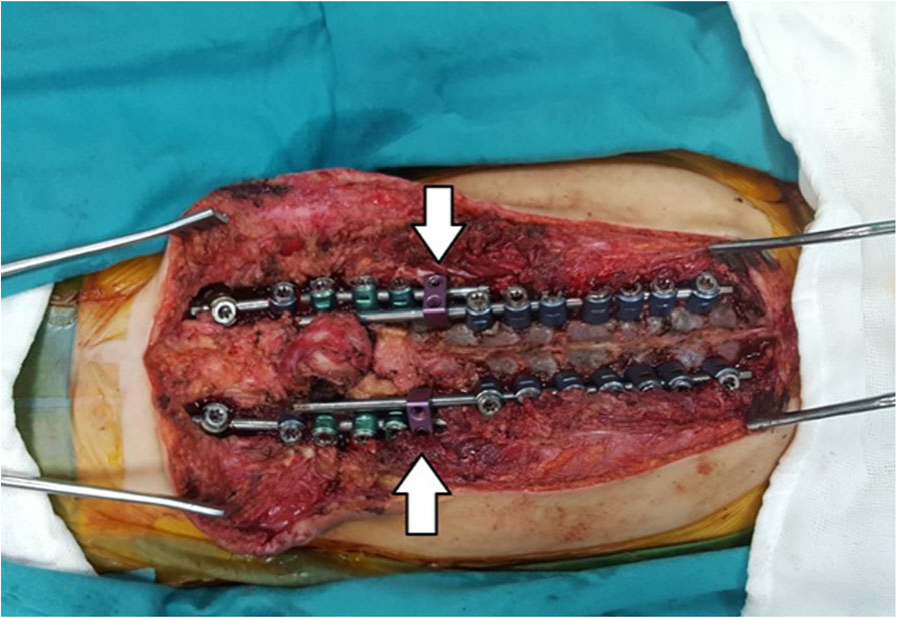
Application of the sliding growing rod (SGR) system. Connection of the SGR to the lumbar kyphectomy and fused area. There will be growth between T4 and T9

**Fig. 3:**
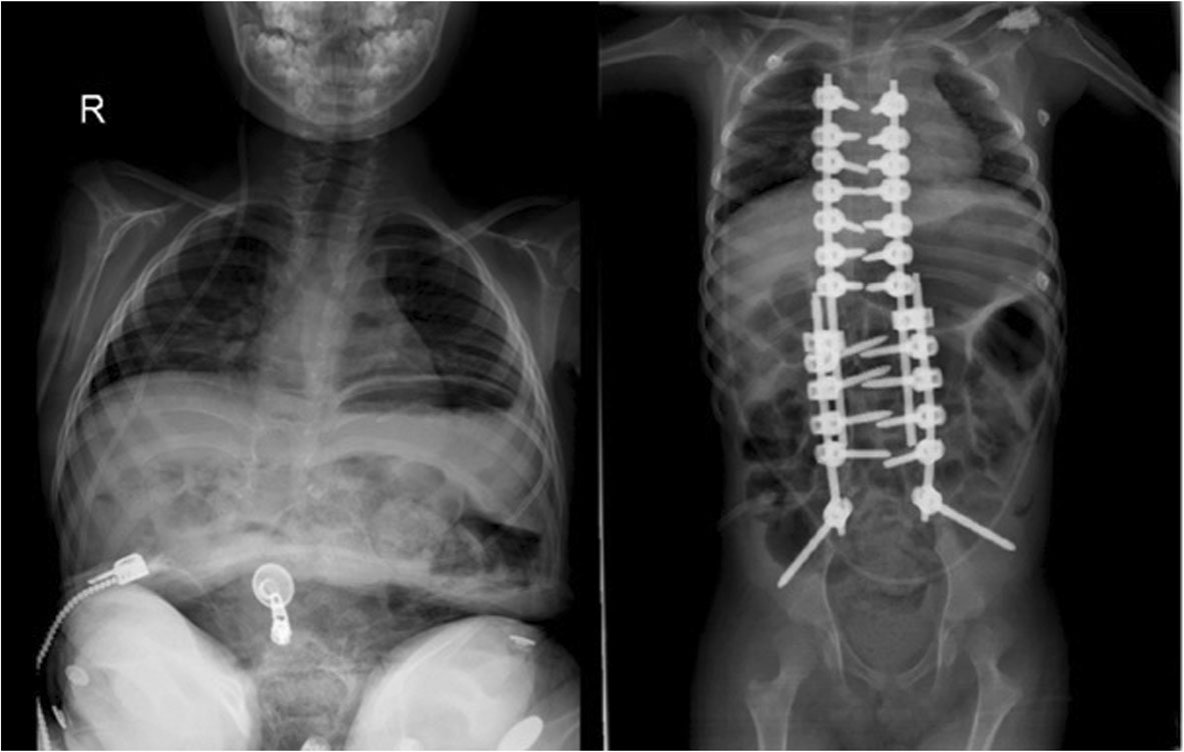
Pre- and early postoperative anteroposterior (AP) radiography

**Fig. 4:**
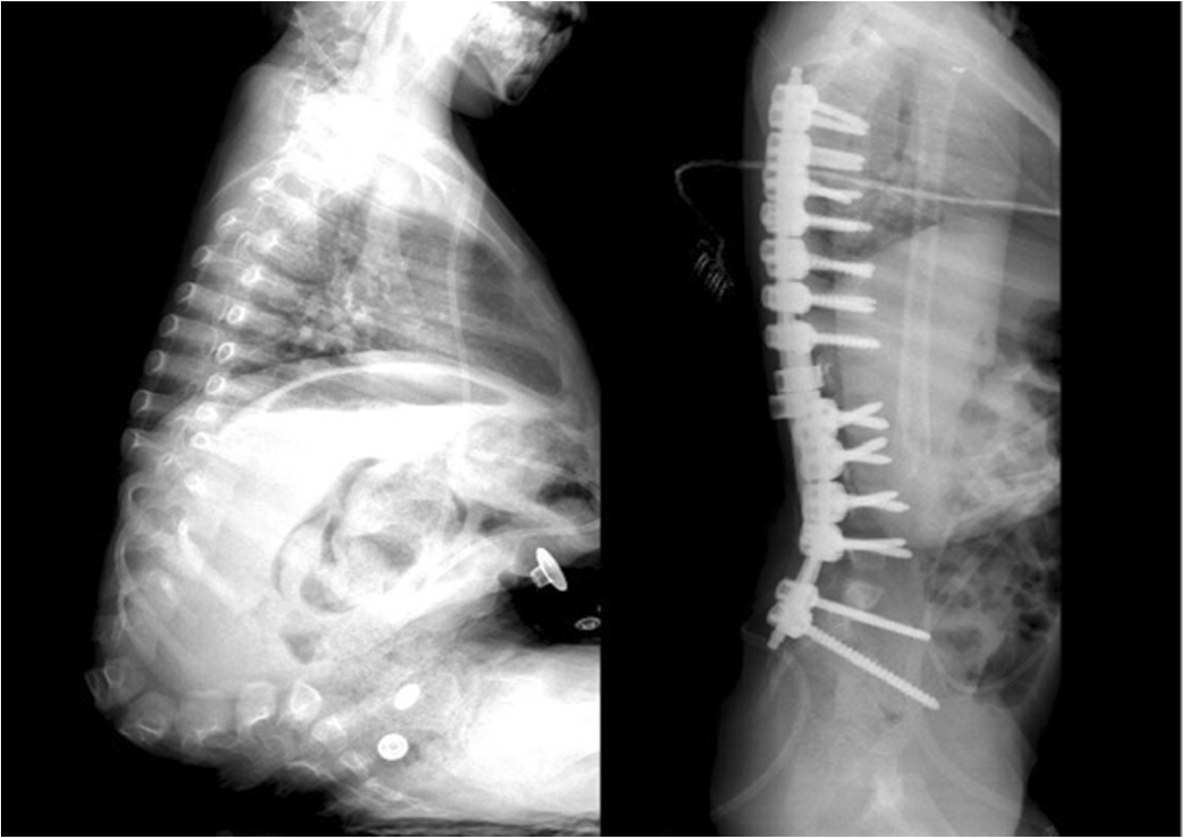
A 5-year-old male patient. Pre- and early postoperative lateral radiography

**Fig 5:**
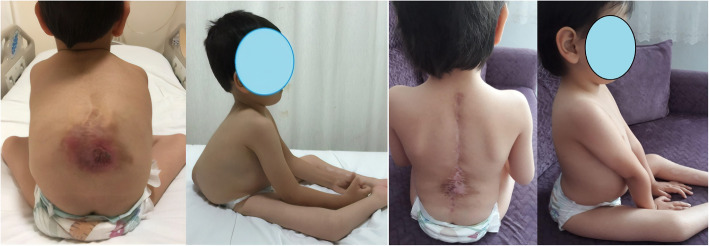
Pre- and postoperative clinical view of the patient

Care was taken when dissecting the corpus anterior part of the deformed vertebrae that caused the kyphosis. The anterior longitudinal ligament (ALL) should be protected and used as a barrier to avoid the vascular bundle in the anterior (Fig. 1). Posterior instrumentation up to T2, T3, or T4 levels was performed in the thoracic region in order to have a strong fixation and a low failure rate. Care was taken to ensure that the iliac pedicle screw was strong. After posterior instrumentation was finished, the kyphectomy was performed while preserving the dura. For bone-to-bone fusion, the remaining proximal and distal vertebral end plates should be resected, and compression should be applied to this area (Fig. 2). The two most proximal pedicle screws and the pedicle screws of the area to be fused were locked to the rod. Nuts of other pedicle screws were left loose. We performed a growth-sparing technique in the area between the two most proximally locked pedicle screws from the kyphectomy area (Figs. 3 and 4). Patients’ incisions were closed without the use of grafts (Fig. 5).

## Materials and methods

We retrospectively analyzed 42 patients with MMC who underwent the SGR technique with kyphectomy and posterior instrumentation between 2016 and 2019. This study was approved by the Umraniye Training and Research Hospital (Number: 00113037880). Inclusion criteria for the study consisted of two main parameters: (1) patients had MMS at T6 and below and (2) patients underwent a kyphectomy during surgery with SGR technique. Patients with a follow-up period of < 1 year, who had undergone previous kyphectomy, and who underwent revision due to failure or pseudarthrosis were excluded from the study.

When the decision was made to apply this surgical technique to MMC patients, we considered patients over the age of 4 years but under the age of 10 with a severe kyphotic deformity that would disrupt the sitting balance or prevent lying on his/her back and also with ulceration or wounds that did not heal at the top of the kyphosis.

Thirty patients who met the study criteria out of 42 patients were retrospectively analyzed. All patients had sensory and motor plegia below the MMC baseline. Twenty-one patients were male, and nine were female. Pre- and postoperative kyphosis and scoliosis, kyphosis and scoliosis correction rates, bleeding amounts during surgery, amount of blood delivered during and after surgery, operation times, instrumentation levels, number of vertebrae resected, MMC onset levels, hospitalization times, and complications were evaluated. Patients’ surgeries were performed by three different spine surgeons. Two spine surgeons always worked together to perform all operations.

X-ray images of the patients were made by a different spinal surgeon who did not perform the surgeries. All patients received anteroposterior (AP) and lateral X-rays in a sitting position before and after surgery [13]. The lengthening of the patients was evaluated by comparing the X-rays in the early postoperative and final evaluations. Lengthening was evaluated by measuring T1–T12 on AP and T1–S1 on lateral X-rays.

Statistical analyses were performed with SPSS 22 (SPSS, IBM, NY, USA). The Mann–Whitney *U* test was used to compare the pre- and postoperative values and nonparametric variables. *P* < 0.05 was considered statistically significant.

## Results

The mean follow-up period of 30 patients was 27.1 (18–40) months. The mean age of the patients during the operation was 6.9 (4–10) days. The mean preoperative kyphosis of the patients was evaluated as 115° (87–166°), the mean early postoperative kyphosis was 3.9° (20–10°), and the final follow-up postoperative kyphosis was 5.1° (22–8°). In nine patients with scoliosis, preoperative scoliosis was evaluated as 60.2° (115–35°). In the early postoperative period, the scoliosis was 12.9° (32–0°). The final follow-up postoperative scoliosis was 15.7° (34–0°). The kyphotic deformity correction rate was 96.5%, and scoliotic deformity correction rate was 74.9%.

The mean number of vertebrae resected during kyphectomy was 2.2 (1–4). The mean surgical time of the patients was 310.2 (200–430) min (Table 1) from the first incision to the last suture. The amount of blood delivered to patients during the surgery and the postoperative period was evaluated as 730 ml (350–1100 ml). The mean hemogram before the operation was 11.2 g/dl (10.3–14.5 g/dl), and that after surgery was 9.1 g/dl (8.0–12.1 g/dl). The mean hospitalization length was 29.5 (7–74) days. Between T1 and T12, the annual prolongation was 0.71 cm/year. When T1–S1 was measured, annual prolongation was evaluated at 0.77 cm/year.

**Table 1:** Reports of 30 patients who underwent kyphectomy for myelomeningocele

Patients	Follow-up	Gender	Age	Preop kyphosis/lordosis	Postop early kyphosis/lordosis	Preop scoliosis	Postop early scoliosis	Instrumentation level	Level of posterior defect	Surgical time (min)	Vertebrae resected	Length of hospital stay	Complication
TT	18	M	4	87	3			T2-iliac	T11	270	1	74	Wound problem, iliac screw revision
MY	20	M	6	133	− 8			T2-iliac	T10	320	3	35	Wound problem, total implant failure
CD	18	E	4	166	− 11			T2-iliac	T11	340	3	45	Wound problem
ZYR	20	F	5	90	0	40	11	T2-iliac	L1	270	3	16	None
MK	24	M	9	89	− 3	45	21	T2-iliac	T12	390	4	14	Wound problem
NB	22	F	5	118	− 1			T2-iliac	T10	300	4	12	Wound problem
ÖB	24	M	4	128	20			T2-iliac	T9	310	1	7	None
AY	40	F	4	96	8			T2-iliac	T10	340	3	43	VAC, 1 debridement.
HAÇ	26	F	10	102	10	41	0	T2-iliac	T10	430	1	47	VAC, 1 debridement.
SK	22	M	10	140	25	60	7	T2-iliac	T8	250	1	45	VAC, 1 debridement.
AÇ	33	M	9	90	5	115	34	T2-iliac	T8	410	3	66	1 debridement, iliac screw remove
MC	32	F	10	131	− 8	53	21	T2-iliac	T10	290	3	20	VAC
TŞ	21	M	5	90	4			T1-iliac	T7	280	2	7	Wound problem
SU	24	F	10	120	− 10			T2-iliac	T8	345	4	7	None
YD	25	M	5	90	2	108	28	T2-iliac	T12	240	1	7	None
DÖ	28	M	4	140	6			T2-iliac	T11	360	2	64	VAC, 1 debridement, need flap
DS	32	M	6	115	24			T4-iliac	T10	240	1	5	None
EA	37	M	13	118	0	35	0	T2-iliac	T9	200	2	48	VAC, 2 debridement,
MA	40	M	9	155	10			T2-iliac	T8	300	3	40	Wound problem, iliac screw revision
MÇ	36	M	6	100	0	45	20	T1-iliac	T7	280	1	45	VAC
ÖÇ	33	M	7	115	3.9			T2-iliac	L1	310	2	32,35	None
ZYR	33	F	6	121	− 3			T2-iliac	T11	330	2	11	None
TY	30	M	9	98	0			T2-iliac	L1	295	1	31	VAC
MO	18	M	8	105	− 7			T2-iliac	T11	335	3	28	PTX
MY	21	M	5	125	9			T2-iliac	T12	350	3	22	Wound problem, ilıac screw remove
ZÖ	25	F	5	90	11			T2-iliac	L1	290	1	23	VAC
İT	29	F	5	127	5			T2-iliac	L1	260	1	13	None
MY	25	M	8	116	14			T2-iliac	T11	305	2	27	VAC
RZ	21	M	10	138	0			T2-iliac	T12	325	3	15	None
MF	36	M	6	119	5			T2-iliac	T10	335	2	38	Wound problem

*M* male, *F* female, *VAC* vacuum-assisted closure, *PTX* pneumothorax

When preoperative kyphosis and postoperative early kyphosis levels of all patients were compared using the Mann–Whitney *U* test, a significant difference between the levels was observed (*p* < 0.05). The postoperative early kyphosis and the kyphosis levels in the final control were compared, and no statistically significant difference was found (*p* > 0.05). In nine patients accompanied by scoliosis, a statistically significant difference was observed when preoperative scoliosis and postoperative early scoliosis values were compared based on the Mann–Whitney *U* test (*p* < 0.05). No statistically significant difference was found when the postoperative scoliosis values were compared with the early postoperative scoliosis values (*p* > 0.05) as shown in Table 2.

**Table 2:** Pre- versus postoperative kyphosis and scoliosis values

	Preoperative	Early postoperative	Final follow-up	*P* value*	*P* value **
Kyphosis	115° (87 to 166)	3.9° (20 to − 10)	5.1° (22 to − 8)	0.000	0.498
Scoliosis	60.2° (115 to 35)	12.9° (32 to 0)	15.7° (34 to 0)	0.000	0.554

*Mann–Whitney *U* test between preoperative and early postoperative kyphosis and scoliosis value

**Mann–Whitney *U* test between early postoperative and final follow-up kyphosis and scoliosis value

### Complications

Postoperative wound problems developed in 19 of 30 patients. A vacuum-assisted closure (VAC) was performed in 10 of 19 patients. Nine patients were followed up with wound dressings. In one of these nine patients, debridement and wound closure were performed in the operating room. The wounds of the other eight patients were healed by the time of follow-up.

VAC treatment for secondary debridement was applied to 10 patients. The culture results from the 10 patients during debridement were positive in eight of these patients. Methicillin-susceptible *Staphylococcus aureus* (MSSA) growth was observed in four patients, and *Escherichia coli* growth was observed in four patients. Antibiotherapy, consisting of ceftriaxone and clindamycin, was started in six patients. One patient received ceftazidime and clindamycin, and one patient only received ceftazidime treatment for 6 weeks. The average VAC treatment in these patients lasted 39.3 (23–64) days. We closed the wound using a gluteus maximus muscle flap in this patient. After VAC treatment, we closed the wounds of the other nine patients without the need for a flap.

Implant failure due to a rod break was observed in one patient. A rod break was seen in the seventh month post-surgically in the area in which kyphectomy was applied. This patient’s 133° kyphosis was reduced to − 8°. After that point, a revision was performed, and the wound problem developed after the revision. The wound was closed on the 27th post-revision day. In the last controls after revision, lumbar kyphosis was measured at − 3°.

Iliac screws were removed in four patients. Two of these patients had iliac screw failure at 4 and 7 months. Only iliac screw revisions were performed in two patients. No problems in any post-revision follow-ups were noted. In another two patients, iliac screws were removed because the rod ends and the head of the iliac pedicle screws had injured the skin.

One patient developed a pneumothorax in the postoperative intensive care unit. A chest tube was attached to this patient, and the chest tube was removed after 6 days of drainage. The pneumothorax was treated without any further complications.

## Discussion

Rigid kyphosis in spinal deformities in patients with MMC seriously complicates their lives. Early treatment always causes easy correction of the deformity and leads to less surgical morbidity in these patients [14]. Especially, since lung development continues until the age of eight post-birth, the progression of kyphosis and scoliosis associated with kyphosis will cause lung problems in these patients in the future and will lead to a more difficult surgical recovery [15].

However, there are some issues discussed in the literature in which long segment fusion performed in early period can cause many problems, such as short stature and decreased lung capacity [16]. Therefore, some studies suggest a short segment fusion to the area where kyphectomy should be performed instead of a long segment fusion. However, a short segment fusion is also an important reason for implant failure in these patients. We need a method that can provide height growth, adequate lung width, and strong stabilization. In fact, after the correction of scoliosis with growth-preserving methods in the early period, this method brought to mind the question of “Why should this method not be done after kyphectomy?”

The developing implant technology in spine surgery and the growing rod technique with the combination of new techniques have started to draw attention in the literature since 2005. Akbarnia et al. used the method of lengthening on double-rod patients using the dual growing rod technique, which actually leads to more application of the growing rod technique in scoliosis patients [17]. In recent years, many methods, such as Shilla and telescopic rods, have been developed as growth-sparing surgery in scoliosis [18]. In the SGR technique, we create a unique locking mechanism which uses both the fixation feature and the slide feature of a polyaxial screw. The system’s design allows for vertebral growth outside the fused apex in the cephalad and caudal directions [19]. Ouyang et al. compared this technique with a posteriorly fused sheep spine to evaluate the stability of the SGR technique in a study conducted in sheep spine. These researchers showed that the SGR technique has stability with respect to flexion, extension, and lateral bending movements in addition to the performance of posterior instrumentation and fusion to the spine [20].

Warner et al. performed kyphectomy in 23 patients with the modified Luque technique in order to maintain height growth in MMC patients [21]. In this technique, he also stated that the lengthening is from the torocal region in the fusion area. In addition, instead of iliac screw stabilization, a Harrington rod was fixed to the sacrum in these patients. The rod was fixed to the sacrum, attached to the dura, and excised. In our study, iliac screw fixation was used instead of sacrum fixation. Also, no need to excise the dura was present. In patients in whom MMC started at lower levels, it is more important to do dura-sparing surgery. In addition, while placing and fixing the rod in the sacrum, the rod can be removed from the bone as anatomical structures in front of the sacrum can be damaged. We think that fixation with a strong iliac screw has less morbidity than sacrum fixation and causes less bleeding. In a study by Can et al., the growing rod and Fackler–Luque techniques were compared in MMC patients. These authors showed a height of 1.05 cm per year between T1 and T12 in patients with growing rods and 0.84 cm per year between T1 and T12 who underwent the Luque technique. According to the study, the growing rod technique emphasized that this technique provides better lengthening than the Luque technique, but surgery should be performed every 6 months for lengthening [5]. In these patients, torocal stability was only achieved by placing pedicle screws at T2 and T3. Continuous surgery for increasing the length of these patients may increase the risks associated with anesthesia in patients. In these studies, patients continuously wore a brace with a thoracolumbosacral orthosis for the first 6 months for stabilization. In our study, we determined that the height growth between T1 and T12 was 0.72 cm per year. Also, if we leave the thoracic rod used for growing in the SGR technique for a long time, the second surgical procedure will can be done at a later period. This timeframe is an advantage of the SGR technique. It is important for stabilization that the torocal fixation is not left to only two vertebrae, so no need to perform the SGR technique exists.

In the literature, complication rates in MMC patients have been reported as very high after kyphectomy. Whether a long segment fusion is performed or the growing rod lengthened, comparison of these techniques also results in 50% complications [5, 22, 23]. The most common complications were wound infections and implant failures. In the 24-case series of Akbar et al., 13 patients had both early and late complications. Implant failure was observed in four of these patients. In this study, patients underwent posterior fusion using Harrington rods and the Warner–Fackler technique [24]. Crank shaft developed in one patient. In a study by Altıok et al. of 33 cases, 17 perioperative complications were seen, and 11 patients underwent revision surgery [25]. In 18 patients of Garg et al., seven minor and six major complications were reported [3]. In 14 cases in a study by Furderer et al., major complications (implant failure and revision surgery) were observed in 10 patients [26]. The most common complications in these studies were implant failures and wound problems. In our study, implant failure due to rod breakage was observed in one patient. Two patients had iliac screw failure. In another two patients, the iliac pedicle screw head injured the skin, creating an ulcer and an unclosed wound in the area, so the iliac screws were removed. Our implant failure rate was lower compared to other studies in the literature. Wound site problems were seen in 19 patients. Our rate of wound problems was similar to that in the literature. Due to the cause of lumbar kyphosis and previous operations in these patients, the skin tissue in this area is of poor quality and very thin. When lumbar kyphosis is converted into lordosis to achieve sagittal balance, a serious gap in this region exists. The hematoma collected in this cavity can cause infection. Therefore, our patients who developed wound problems were re-granulated with VAC treatment in the early period. VAC treatment is an important tool for the treatment of infection seen after spinal deformity surgery in children. VAC treatment applied in this study showed successful results [27].

The limitations of this study were the inclusion of only a small number of patients and the retrospective nature of the study. In the literature, the number of cases in MMC patients is generally small in articles concerning the kyphectomy technique. Therefore, we think that the number of cases in our study is the same as the articles in the literature. In the literature, an article using the SGR technique with kyphectomy in MMC patients has not been published yet.

Many surgical techniques have been described in MMC patients concerning kyphectomy. In these patients, achieving a balance between stabilization and growth-preserving techniques was attempted. Adequate stabilization for fusion in the SGR technique can be achieved in these patients. The expected increase in height at the yearly follow-ups can also be achieved. We think performance of SGR technique after kyphectomy is more reliable and effective in MMC patients.

## Abbreviations

SGR: Sliding growing rod
MMC: Myelomeningocele
AP: Anteroposterior
ALL: Anterior longitudinal ligament
VAC: Vacuum-assisted closure
MSSA: Methicillin-susceptible *Staphylococcus aureus*
